# GITR Intrinsically Sustains Early Type 1 and Late Follicular Helper CD4 T Cell Accumulation to Control a Chronic Viral Infection

**DOI:** 10.1371/journal.ppat.1004517

**Published:** 2015-01-15

**Authors:** Derek L. Clouthier, Angela C. Zhou, Michael E. Wortzman, Olga Luft, Gary A. Levy, Tania H. Watts

**Affiliations:** 1 Department of Immunology, University of Toronto, Toronto, Ontario, Canada; 2 University of Toronto Transplantation Institute, Toronto, Ontario, Canada; 3 Department of Laboratory Medicine and Pathobiology, Faculty of Medicine, University of Toronto, Toronto, Ontario, Canada; Nationwide Children’s Hospital, UNITED STATES

## Abstract

CD4 T cells are critical for control of persistent infections; however, the key signals that regulate CD4 T help during chronic infection remain incompletely defined. While several studies have addressed the role of inhibitory receptors and soluble factors such as PD-1 and IL-10, significantly less work has addressed the role of T cell co-stimulatory molecules during chronic viral infection. Here we show that during a persistent infection with lymphocytic choriomeningitis virus (LCMV) clone 13, mice lacking the glucocorticoid-induced tumor necrosis factor receptor related protein (GITR) exhibit defective CD8 T cell accumulation, increased T cell exhaustion and impaired viral control. Differences in CD8 T cells and viral control between GITR^+/+^ and GITR^-/-^ mice were lost when CD4 T cells were depleted. Moreover, mixed bone marrow chimeric mice, as well as transfer of LCMV epitope-specific CD4 or CD8 T cells, demonstrated that these effects of GITR are largely CD4 T cell-intrinsic. GITR is dispensable for initial CD4 T cell proliferation and differentiation, but supports the post-priming accumulation of IFNγ^+^IL-2^+^ Th1 cells, facilitating CD8 T cell expansion and early viral control. GITR-dependent phosphorylation of the p65 subunit of NF-κB as well as phosphorylation of the downstream mTORC1 target, S6 ribosomal protein, were detected at day three post-infection (p.i.), and defects in CD4 T cell accumulation in GITR-deficient T cells were apparent starting at day five p.i. Consistently, we pinpoint IL-2-dependent CD4 T cell help for CD8 T cells to between days four and eight p.i. GITR also increases the ratio of T follicular helper to T follicular regulatory cells and thereby enhances LCMV-specific IgG production. Together, these findings identify a CD4 T cell-intrinsic role for GITR in sustaining early CD8 and late humoral responses to collectively promote control of chronic LCMV clone 13 infection.

## Author Summary

The natural rodent pathogen LCMV clone 13 causes a persistent viral infection in mice and has successfully predicted several immunological factors that are relevant to human chronic viral infection such as HIV. LCMV clone 13 infection is ultimately controlled by cell-mediated and humoral immune responses by day 60–90 post-infection in CD4 T cell-sufficient mice. While it has been known for several years that CD4 T cell help is critical for control of LCMV clone 13, research to date has been largely limited to the regulatory factors that contribute to late CD4 T cell dysfunction, with little knowledge of the role of T cell co-stimulatory factors in sustaining CD4 T cells to help cell-mediated and humoral immune responses. Using GITR-deficient mice, we show that the co-stimulatory molecule GITR plays a critical cell-intrinsic role in early CD4 T cell accumulation to support cytotoxic T cell responses and late LCMV-specific IgG production. The early effects of Th1 on CTL responses are IL-2-dependent. Mice lacking GITR have up to 35-fold higher viral burden relative to GITR-sufficient controls. Taken together, we demonstrate a critical cell-intrinsic role for GITR is sustaining CD4 T cell responses to control chronic LCMV infection. Thus, GITR on CD4 T cells may critically contribute to the initial viral set-point in infections such as HIV.

## Introduction

During chronic viral infections, exemplified by the clone 13 variant of lymphocytic choriomeningitis virus (LCMV cl 13), persistent antigen presentation results in the functional exhaustion of the T cell response, characterized by persistent upregulation of inhibitory molecules and a progressive loss of T cell effector functions [1]. Ultimately, LCMV cl 13 is cleared by 60–90 days p.i. due to both T- and B-cell responses. While there have been a number of studies on the role of T cell inhibitory receptors and anti-inflammatory cytokines during chronic infection [2–7], rather less has been done to study the role of co-stimulatory receptors in this context. Co-stimulatory TNFR family members are of particular interest in this regard because they are often induced upon antigen receptor signaling, leading to their co-expression with inhibitory receptors during a persistent infection [8–10]. CD4 T cell help is critical for the control of chronic infections. The removal of CD4 T cells from mice prior to infection with LCMV cl 13 [11–13], or the loss of CD4 T cells during progressive HIV infection [14] leads to increased viral burden, immune dysregulation, and functional T cell exhaustion. While CD4 cells are clearly implicated in the control of chronic viral infections, the co-stimulatory signals that contribute to CD4 T cell help remain poorly defined. Evidence to date suggests that there is significant heterogeneity in the potency and mechanisms of T cell modulation by members of the TNFR superfamily during chronic viral infection [8, 9, 15–18].

The Glucocorticoid-induced TNFR related protein (GITR) and its ligand (GITRL) are induced upon activation of a number of immune cell types [19]. GITR is expressed at low levels on resting T cells, but its expression rapidly increases upon activation. Although constitutively expressed on Foxp3^+^ regulatory T cells (Treg), GITR is dispensable for Treg function but can play a role in the accumulation of both regulatory and effector T cells *in vivo* [20, 21]. In many experimental systems, it has been difficult to determine the key cell types through which GITR mediates its effects. Controversy over the relative role of GITR on effector versus regulatory T cells persists, though these effects may be context-dependent [19, 22, 23]. GITR-deficient mice are viable, reproduce normally, and have no obvious immunological defects in the naïve state [24]. Studies using GITR-deficient mice or an agonist anti-GITR antibody have shown an immune stimulatory role for GITR in the context of viral infections, but the mechanisms underlying these phenomena are not entirely clear [19].

Here we address the role of GITR during chronic viral infection using GITR-deficient mice infected with LCMV cl 13 [25]. We find that GITR^-/-^ mice have 2–3-fold fewer LCMV-specific CD8 T cells post-priming and throughout the infection, with concomitant increases in viral load. A previous study showed that GITR on TCR transgenic CD8 T cells contributes to the control of influenza infection [26]. Surprisingly however, during LCMV cl 13 infection, the effect of GITR was largely CD4 T cell-intrinsic and independent of Tregs. GITR-deficiency impaired the accumulation of IFNγ^+^IL-2^+^ T helper 1 (Th1) cells as well as follicular helper T cells (Tfh) and the production of LCMV-specific IgG. Mixed bone marrow chimeras and adoptive transfer studies of LCMV-specific CD4 and CD8 T cells demonstrate that the effects of GITR are CD4 T cell-intrinsic. Neutralization of IL-2 in GITR^+/+^ mice reduced the immune response to the level observed in GITR^-/-^ mice, whereas IL-2 neutralization in GITR^-/-^ mice had no effect, revealing a central role for this cytokine in GITR-dependent control of LCMV cl 13. Taken together, our studies provide evidence for a critical role for GITR in the post-priming accumulation of IL-2^+^ Th1 cells to help cell-mediated immunity as well as Tfh accumulation to sustain the humoral arm of the immune response to control a persistent viral infection.

## Results

### GITR is required for CD8 T cell accumulation and function post-priming and control of chronic LCMV infection

**Figure 1:**
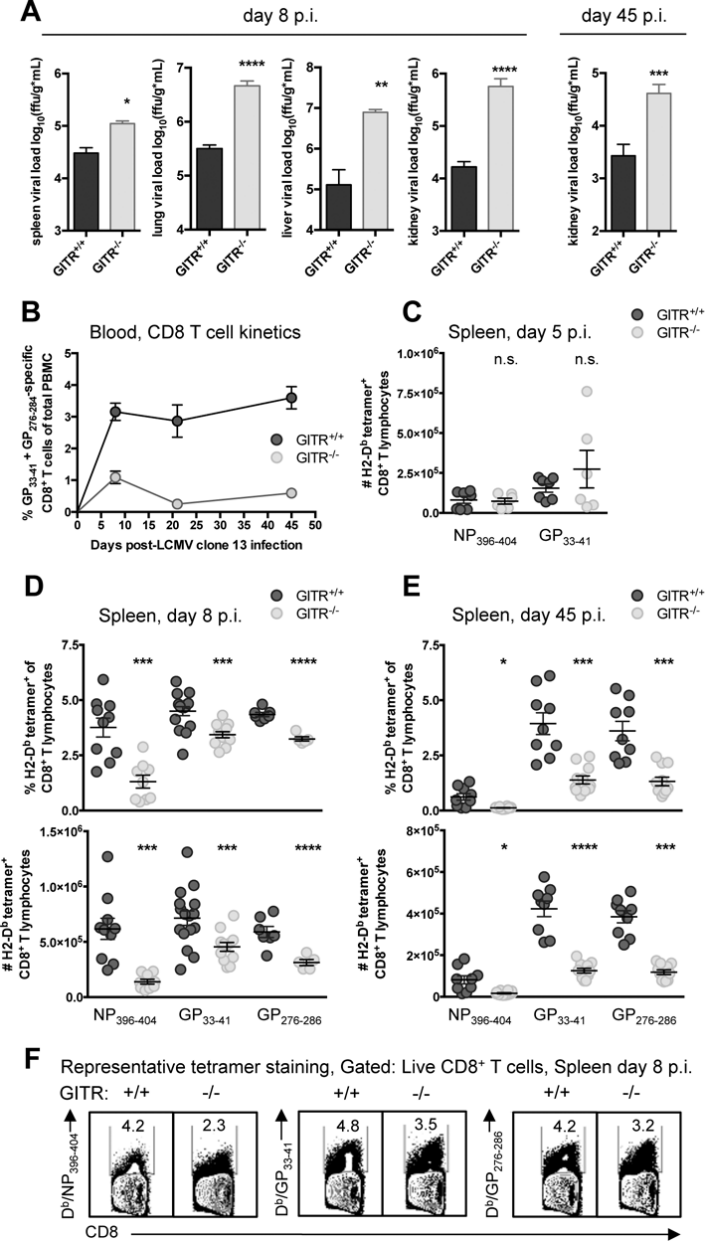
GITR^-/-^ mice have impaired CD8 T cell responses and compromised control of chronic LCMV cl 13 infection. (A) GITR^+/+^ and GITR^-/-^ mice were infected i.v. with 2×10^6^ ffu of LCMV cl 13 and viral load was measured in spleen, lung, liver, and kidney of mice at day eight p.i. and kidney at day 45 p.i. (B) LCMV-specific (pooled D^b^/GP_33–41_+GP_276–286_) T cell responses in peripheral blood were assessed by flow cytometry from day eight to 45 p.i. (C) Absolute numbers of D^b^/NP_396–404_- and D^b^/GP_33–41_-specific CD8^+^ T cells are shown in the spleen from day five p.i. (D, E) Frequencies and absolute numbers of D^b^/NP_396–404_-, D^b^/GP_33–41_-, and D^b^/GP_276–286_-specific cells are shown in the spleen from day eight p.i. and 45 p.i. (F) Representative flow cytometry data showing detection of LCMV-tetramer specific CD8 T cells from spleens at day eight p.i. Bar plots in A represent mean ± SEM and are pooled from at least two independent experiments of at least four mice per group. Each symbol in B shows mean ± SEM of at least three mice per group, representative of at least two experiments per time point. Each symbol in C–E represents an individual mouse, with bars indicating mean ± SEM, from at least two independent repeats, with at least four mice per group.

To evaluate the role of GITR in a chronic infection, we infected wild-type mice (GITR^+/+^) or mice with ablated *Tnfrsf18* (GITR^-/-^) [24] with LCMV cl 13. At both acute and chronic time points (days eight and 45 p.i.), GITR^-/-^ mice had significantly increased viral load (Fig. 1A). These effects were particularly striking in the kidney, where GITR^-/-^ mice had 35-fold higher viral load than GITR^+/+^ mice at day eight p.i. GITR^-/-^ mice had substantially fewer LCMV-specific CD8 T cells in peripheral blood (Fig. 1B, for gating strategy see S1 Fig.). Following initial infection and T cell priming at day five p.i., there was no difference in the number of splenic LCMV-specific CD8 T cells between GITR^+/+^ and GITR^-/-^ mice (Fig. 1C). However, between days five and eight p.i., a significant impairment in the frequency and total number of splenic LCMV-specific CD8 T cells in GITR^-/-^ mice materializes, and this effect becomes even more striking at day 45 p.i. (Fig. 1, D–F).

**Figure 2:**
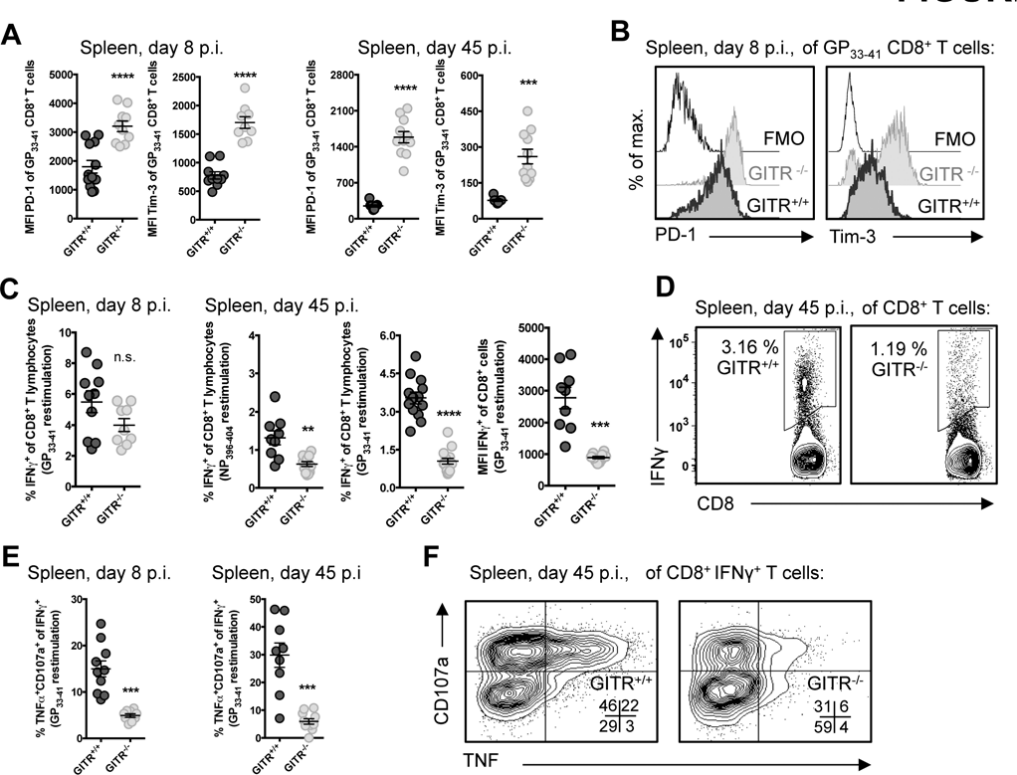
GITR^-/-^ LCMV-specific CD8 T cells express higher levels of inhibitory molecules and are more functionally exhausted. (A, B) GITR^+/+^ and GITR^-/-^ mice were infected as in Fig. 1. Expression of PD-1 and Tim-3 were measured on LCMV-specific CD8 T cells at day eight and 45 p.i., with representative histograms shown for GP_33–41_-specific CD8 T cells from day eight p.i. (C-F) Splenocytes from day eight and 45 p.i. were restimulated with D^b^-restricted LCMV peptides (as indicated) after which intracellular cytokines and surface CD107a were measured by flow cytometry, with summary data and representative flow cytometry plots shown. All staining panels are from GP_33–41_-specific responses, with similar data from NP_396–404_ and GP_276–286_. Each symbol represents an individual mouse, with bars indicating mean ± SEM. All parameters were measured in at least two of three repeats, with at least four mice per group.

Co-signaling molecules PD-1 and Tim-3 impair CD8 T cell responses to LCMV cl 13 [4, 5]. The majority of the LCMV-specific CD8 T cells in GITR^+/+^ and GITR^-/-^ mice were PD-1^+^ Tim-3^+^ after LCMV cl 13 infection, but CD8 T cells from GITR^-/-^ mice expressed significantly higher levels of PD-1 and Tim-3 per cell at days eight and 45 p.i. (Fig. 2, A and B). CD8 T cells from GITR^+/+^ or GITR^-/-^ mice produced similar amounts of IFNγ following peptide restimulation at day eight p.i., but by day 45 p.i., the GITR^-/-^ mice had fewer IFNγ^+^ CD8 T cells with lower IFNγ production per cell (Fig. 2, C and D). Moreover, at all time points analyzed, fewer of the IFNγ-producing CD8 T cells from GITR^-/-^ mice exhibited a multifunctional phenotype as defined by TNF and surface CD107a expression (a surrogate marker for cellular cytotoxic activity) (Fig. 2, E and F). These findings reveal a key role for GITR in the CD8 T cell response and control of chronic LCMV infection. We also examined the role of GITR with the acutely infecting Armstrong variant of LCMV and observed approximately two- to three-fold more GP_33–41_-specific CD8 T cells in GITR^+/+^ compared to GITR^-/-^ at day eight p.i. (p<0.01 in two of the three experiments, total of ten mice per group), however both GITR^+/+^ and GITR^-/-^ mice cleared LCMV Armstrong infection by day eight p.i.

### GITR potentiates the CD8 T cell response through CD8 T cell-extrinsic effects

**Figure 3:**
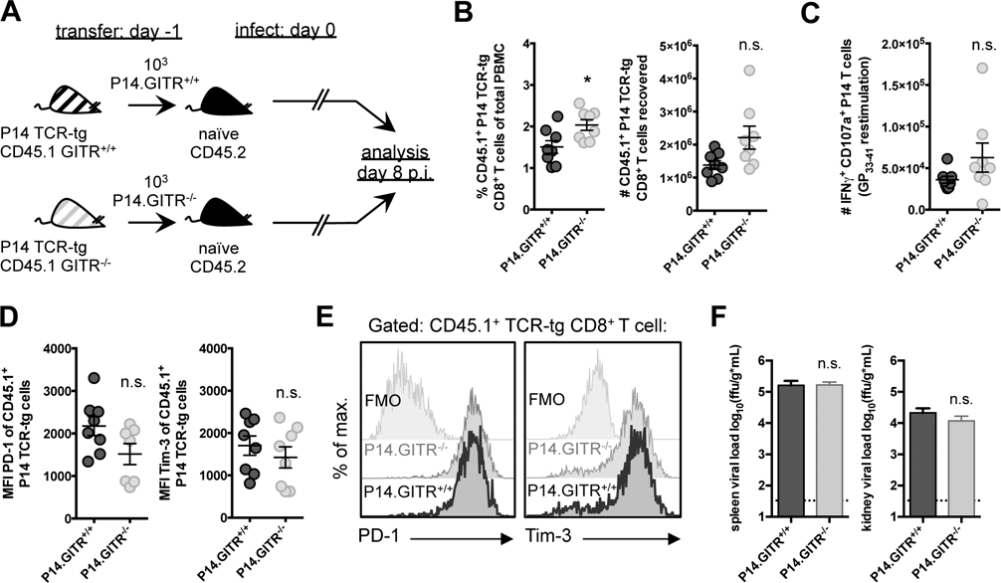
The effect of GITR on the CD8 T cell response is CD8 T cell-extrinsic. (A) Schematic indicating that 10^3^ GITR^+/+^ or GITR^-/-^ CD45.1 P14 cells from F2 littermates were adoptively transferred into separate naïve CD45.2 congenic mice one day prior to infection with 2×10^6^ ffu LCMV cl 13. (B and C) At day eight p.i., the frequency of CD45.1 P14 cells in peripheral blood (B, left) and the number of total (B, right) and IFNγ^+^CD107a^+^TNF^+^ (C) P14 cells were determined. (D, E) CD45.1 P14 cells were evaluated for expression of PD-1 and Tim-3, with summary plots and representative stains shown. (F) Viral load in spleen and kidney was evaluated from mice at day eight p.i. Broken horizontal lines represent the limit of detection. Each symbol represents an individual mouse, with bars indicating mean ± SEM. Data are representative of four experiments with at least four mice per group, with two repeats in the blood and two repeats in the spleen and kidney.

Previous work has shown an intrinsic role for GITR in sustaining the survival of TCR transgenic CD8 T cells during acute influenza virus infection [26]. To determine if CD8 T cell-intrinsic effects were also responsible for defects in LCMV cl 13 control in GITR^-/-^ mice, we crossed GITR^-/-^ with P14 mice, which have a transgenic TCR specific for the D^b^-restricted LCMV GP_33–41_ epitope [27]. We transferred 10^3^ purified CD8 T cells from CD45.1 GITR^+/+^ or GITR^-/-^ P14 littermates into CD45.2 congenic mice one day prior to LCMV cl 13 infection (Fig. 3A). P14 T cells lacking GITR showed no impairment in expansion, and in fact showed a slight increase in frequency (Fig. 3B). There was also no difference in IFNγ production or degranulation, PD-1 or Tim-3 expression between the GITR-sufficient or GITR-deficient P14 T cells (Fig. 3, C–E). Moreover, the absence of GITR only on the transferred P14 T cells had no effect on viral control at day eight p.i. (Fig. 3F). Thus, the effect of GITR in potentiating the CD8 T cell response to LCMV cl 13 is CD8 T cell-extrinsic.

### GITR critically regulates early Th1 responses to LCMV

**Figure 4:**
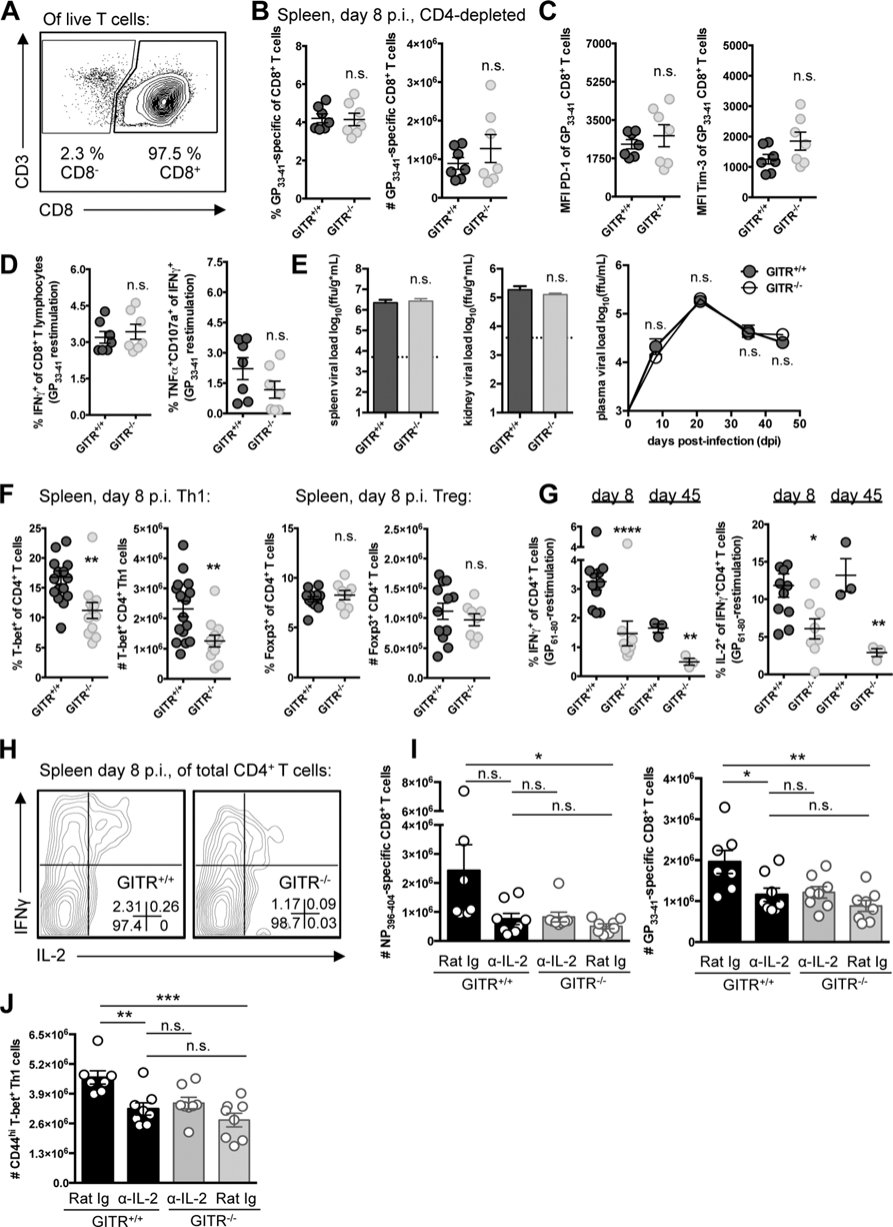
A CD4 T cell population underscores the defective immunity in GITR^-/-^ mice, and GITR^-/-^ mice have fewer IL-2-producing Th1 cells. (A) Mice were administered a single 0.5mg i.p. dose of anti-CD4 two days prior to infection with LCMV cl 13 and the level of CD4 depletion evaluated in the spleen by flow cytometry at day eight p.i. (B) At day eight p.i., the frequency and absolute number of CD8 T cells that were GP_33–41_-specific in GITR^+/+^ and GITR^-/-^ CD4-depleted mice was determined. (C, D) PD-1 and Tim-3 expression, and functionality in terms of IFNγ production and CD107a/TNF co-production by CD8 T cells after LCMV GP_33–41_ peptide restimulation. (E) Viral load was evaluated between CD4-depleted GITR^+/+^ and GITR^-/-^ spleen and kidney at day eight p.i. (bar graphs) as well as from day eight to 45 p.i. in the serum (right). Horizontal broken lines represent the limit of detection. (F) GITR^+/+^ and GITR^-/-^ F2 littermates were infected as in Fig. 1, and the absolute number of CD4 T cells and the proportion and number of these cells expressing T-bet or Foxp3 at day eight p.i. (G, H) Freshly isolated splenocytes were restimulated with the I-A^b^-restricted LCMV GP_61–80_ peptide and IFNγ^+^ and IFNγ^+^IL-2^+^ cells were evaluated at days eight and 45 p.i., with representative staining shown. (I, J) GITR^+/+^ and GITR^-/-^ mice were given 0.5mg i.p. anti-IL-2 or isotype control on days four and six p.i., and the absolute numbers of D^b^/NP_396–404-_ and D^b^/GP_33–41_-specific CD8^+^ T cells and Th1 cells from spleen are shown. Each symbol represents an individual mouse, with bars indicating mean ± SEM. For A–E, data are pooled from spleens from two experiments with at least three mice per group per experiment, with a third repeat in peripheral blood showing similar results. Plasma viral load is shown for one experiment with three mice per group. For F–H, data from day eight p.i. are pooled from spleens from three experiments with at least four mice per group per experiment and data from day 45 p.i. is a total of three mice per group. Data in I–J are from two experiments with at least seven mice per group.

To evaluate the importance of CD4 T cells in this model, we depleted CD4 cells from GITR^+/+^ and GITR^-/-^ mice prior to LCMV cl 13 infection (Fig. 4A). CD4-depleted GITR^+/+^ mice had fewer and less functional LCMV-specific CD8 T cells, and 100-fold higher viral load relative to non-CD4-depleted GITR^+/+^ mice, consistent with previous reports of CD4 depletion increasing the severity of LCMV cl 13 infection [11–13]. However, the qualitative and quantitative differences between GITR^+/+^ and GITR^-/-^ CD8 T cell responses and viral control at day eight p.i. were largely lost in CD4-depleted mice (Fig. 4, B–E), suggesting that CD4 T cells are necessary for the effect of GITR on the CD8 T cell response to LCMV cl 13 as well as viral control.

We next analyzed the effect of GITR on CD4 T cell responses. GITR^-/-^ mice had similar total numbers of CD4 T cells in the spleen following LCMV infection at day eight p.i. However, fewer of the GITR^-/-^ CD4 T cells expressed the signature Th1 transcription factor T-bet (Fig. 4F, for gating strategy see S2 Fig.) and there was a striking three-fold reduction in the percent of CD4 T cells that were IFNγ^+^ or IFNγ^+^IL-2^+^ following GP_61–80_-restimulation at day eight p.i., a defect that was maintained out to day 45 p.i. (Fig. 4, G and H). In contrast, GITR^+/+^ and GITR^-/-^ littermates had similar proportions and absolute numbers of Foxp3^+^ Tregs (Fig. 4F). Moreover, depletion of Tregs did not recapitulate the effects of total CD4 T cell depletion on the CD8 T cell response (S3 Fig.).

### IL-2 is necessary for the effect of GITR on CD8 T cell responses

IL-2 is important for CD8 T cell accumulation and function in the context of chronic LCMV infection [28–30]. Therefore, we evaluated whether the effects of GITR on the T cell response were dependent on IL-2. Of note, we did not detect any IL-2^+^ CD8 T cells after *ex vivo* peptide stimulation at day eight p.i., consistent with previous reports [31]. As the CD8 T cell deficit in GITR^-/-^ mice is not realized until after day five p.i., we administered 0.5mg of blocking anti-IL-2 to mice on days four and six p.i., and evaluated T cell responses at day eight p.i. IL-2 blockade reduced the LCMV-specific Th1 and CD8 T cell responses in GITR^+/+^ mice to the level observed in GITR^-/-^ mice, whereas IL-2 blockade had no impact on the T cell responses in GITR^-/-^ mice (Fig. 4, I and J). Thus, GITR is critical for Th1 responses and IL-2-dependent help for CD8 T cells.

### GITR enhances follicular helper CD4 T cell responses to LCMV cl 13 and the production of LCMV-specific IgG

**Figure 5:**
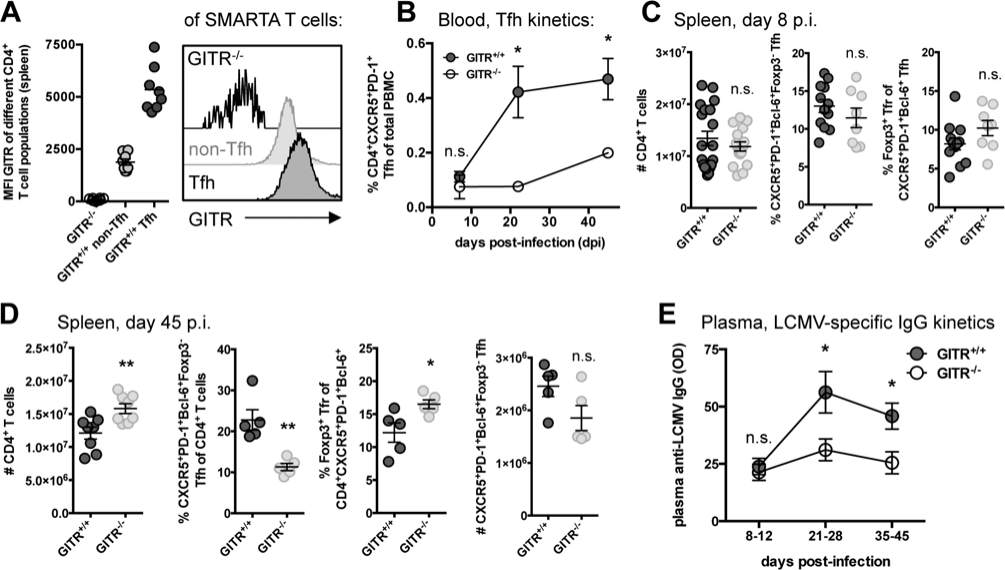
GITR^-/-^ mice have defective follicular helper CD4 T cell responses following LCMV cl 13 infection. (A) Naïve C57BL/6 mice received 10^4^ cells of a 1:1 mix of GITR^+/+^ and GITR^-/-^ SMARTA and were infected the following day with LCMV cl 13. The expression of GITR on SMARTA Tfh and non-Tfh was determined at day eight p.i., with representative staining shown. (B) The frequency of Tfh in peripheral blood of non-TCR transgenic GITR^-/-^ or GITR^+/+^ mice was monitored from day seven to 45 p.i. Each symbol represents a total of at least six mice per group, from two independent experiments. (C, D) The absolute number of total CD4 T cells and the frequency of Tfh and Tfr were determined in GITR^+/+^ and GITR^-/-^ mice at day eight p.i. (C) and day 45 p.i. (D); total numbers of Tfh were also determined at day 45 p.i. (D). (E) Total plasma LCMV-specific IgG response was monitored in GITR^+/+^ and GITR^-/-^ mice by ELISA between day eight and 45 p.i., each symbol represents a total of at least six mice per group, from at least two experiments from each time point. OD values are reported from the 1:50 dilution, which was in the linear range of the titration curve. Unless otherwise noted, each symbol represents an individual mouse, with bars indicating mean ± SEM. Data in A are pooled from two independent experiments with at least four mice per group. Data in C are pooled from at least eight mice per group, from at least two independent experiments. Data in D are pooled from two independent experiments with two to three mice per group.

GITR impacts viral load both early and late during LCMV cl 13 infection (Fig. 1A). As Tfh have emerged as an important CD4 T cell subset that promotes late B cell responses to LCMV cl 13 infection [32, 33], we examined the effects of GITR on Tfh. At day eight p.i., we noted elevated levels of GITR expression on Tfh compared to non-Tfh CD4^+^ SMARTA cells (SMARTA mice express a transgenic TCR specific for the I-A^b^-restricted LCMV GP_61–80_) (Fig. 5A) [34]. Evaluation of the Tfh response in peripheral blood of GITR^+/+^ and GITR^-/-^ mice between day seven and 45 p.i. indicated a significantly lower frequency of Tfh in GITR^-/-^ mice compared to GITR^+/+^ mice at late (days 21 and 45 p.i.) but not early times during the response to LCMV cl 13 (Fig. 5B). T follicular regulatory cells (Tfr) are Foxp3^+^ T cells that localize to germinal centers to regulate the response [35, 36]. The spleens of GITR^+/+^ and GITR^-/-^ mice had similar proportions of Tfh and Tfr at day eight p.i., but at day 45 p.i. GITR^-/-^ mice exhibited significantly fewer Tfh and there was a significant increase in the proportion of Tfr in GITR^-/-^ compared to GITR^+/+^ mice (Fig. 5, C and D). Moreover, GITR^-/-^ mice had significant impairment in the LCMV-specific IgG response at day 21–45 p.i. (Fig. 5E).

### GITR acts intrinsically on CD4 T cells

**Figure 6:**
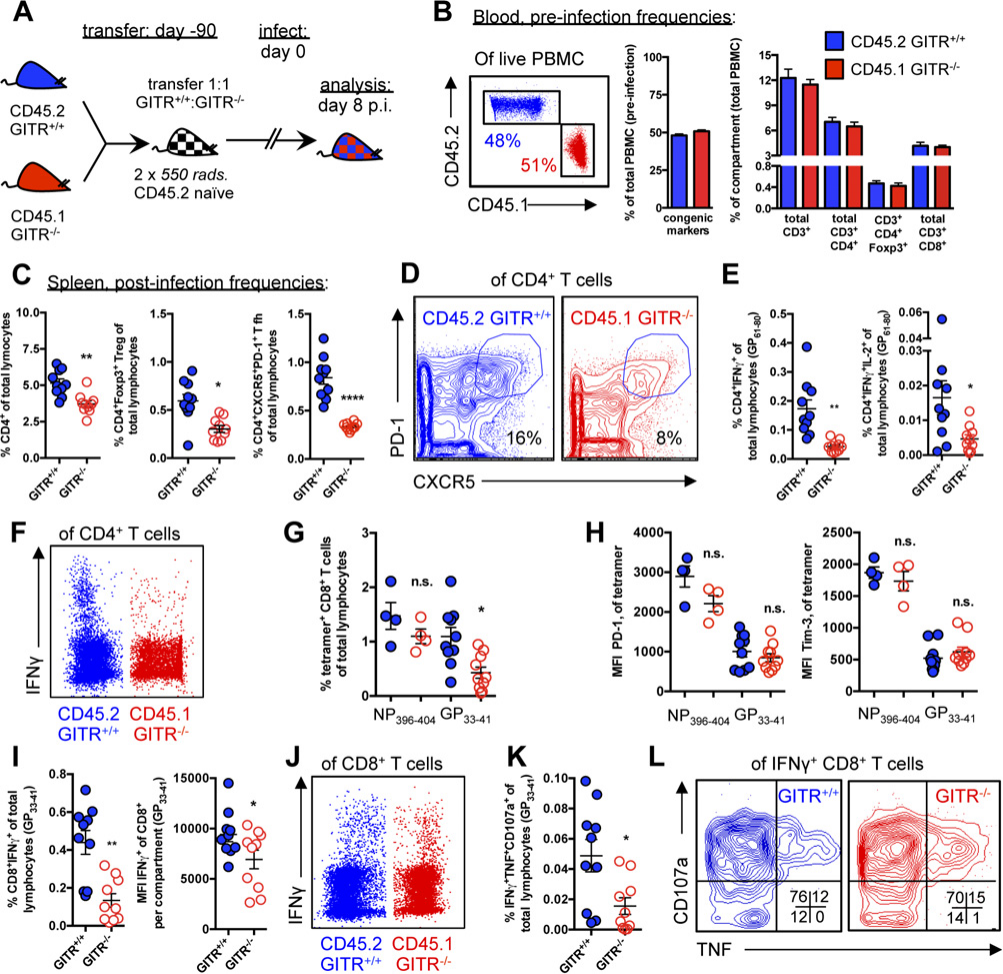
The effects of GITR-deficiency on immunity to LCMV are largely CD4 T cell-intrinsic. (A) Schematic indicating that lethally irradiated age- and sex-matched C57BL/6 mice were reconstituted with a 1:1 mixture of CD45.2 GITR^+/+^: CD45.1 GITR^-/-^ bone marrow cells. (B) At 90 days post-reconstitution, mice were bled to evaluate the proportion of GITR^+/+^ (CD45.2) and GITR^-/-^ (CD45.1) total, CD8, CD4, and CD4 Foxp3^+^ cells prior to infection as in Fig. 1. (C, D) The proportions of GITR^+/+^ and GITR^-/-^ total CD4, Foxp3^+^ Treg, and CXCR5^+^ PD-1^hi^ Tfh were determined at day eight p.i., with representative staining shown for Tfh. (E, F) The proportions of IFNγ^+^ or IFNγ^+^IL-2^+^ GITR^+/+^ and GITR^-/-^ CD4 T cells were evaluated following five hours of I-A^b^-restricted LCMV GP_61–80_ peptide restimulation, with representative staining shown. (G, H) The frequencies of GITR^+/+^ and GITR^-/-^ D^b^/NP_396–404-_ and D^b^/GP_33–41_-specific CD8 T cells, and the expression of PD-1 and Tim-3 in the spleen were evaluated at day eight p.i. (I, J) Freshly isolated splenocytes from chimeric mice sacrificed at day eight p.i. were restimulated with D^b^-restricted LCMV GP_33–41_ peptide for five hours in the presence of Brefeldin A and monensin, after which intracellular cytokines and surface markers were measured by flow cytometry, with representative staining shown. (K, L) Co-production of TNF and CD107a by the IFNγ^+^ population was evaluated, with representative staining shown. Each symbol represents an individual mouse, with bars indicating mean ± SEM. Data are pooled from two experiments with a total of ten mice, for all parameters except NP_396–404_, which was only evaluated in one experiment of four mice.

Although CD4 T cells are required for the effect of GITR on the CD8 T cell response to LCMV, it was conceivable that GITR on another cell population could indirectly affect CD4 T cell help [19]. To evaluate a cell-intrinsic effect of GITR, we used mixed bone marrow chimeras, in which GITR^+/+^ and GITR^-/-^ cells compete in the same mouse. If GITR intrinsically affects a specific cell type, then the GITR^-/-^ cells will be at a competitive disadvantage, whereas if the effect of GITR is extrinsic then GITR^+/+^ and GITR^-/-^ cells would be equally affected by the lack of GITR in another cell compartment and their ratio would be constant. To this end, we reconstituted lethally irradiated C57BL/6 mice with 1:1 mixture of CD45.2 GITR^+/+^:CD45.1 GITR^-/-^ bone marrow cells (Fig. 6A). Although we used CD45.2 recipients, we have found that in separate control experiments using identical radiation schedules, >95% of lymphocytes were of donor origin at three months post-reconstitution. Consistent with the minimal contribution from residual T cells following lethal irradiation, we verified that the CD45.2:CD45.1 ratio in peripheral blood was 1:1, with similar proportions of CD4, CD8 T cells and Foxp3^+^ Tregs prior to infection (Fig. 6B).

At day eight p.i., there was a slight but significant effect of GITR on total CD4 cells, with fewer CD4 T cells in the GITR^-/-^ compartment (Fig. 6C). Among the CD4 cells, there was a two-fold reduction in GITR^-/-^ Tregs and a three-fold reduction in GITR^-/-^ Tfh (Fig. 6, C and D). Additionally, there was a three-fold reduction in IFNγ-producing GITR^-/-^ Th1 cells after LCMV GP_61–80_ restimulation, of which fewer co-produced IL-2 (Fig. 6, E and F). Taken together, these data demonstrate a key CD4 T cell-intrinsic role of GITR during LCMV cl 13 infection, with the most dramatic effects on the Th1 and Tfh responses.

At day eight p.i., there was no difference in the frequency of GITR^+/+^ and GITR^-/-^ NP_394–404_-specific CD8 T cells, whereas the frequency of GITR^-/-^ GP_33–41_-specific T cells was significantly reduced compared to the GITR^+/+^ population (Fig. 6G), however in contrast to the complete knockout mouse (Fig. 2), the levels of PD-1 and Tim-3 were similar between the GITR^+/+^ and GITR^-/-^ compartments (Fig. 6H). The frequency of CD8 T cells with the ability to produce cytokines and degranulate after GP_33–41_ peptide restimulation was also decreased in GITR^-/-^ compared to GITR^+/+^ (Fig. 6, I–L). These data show that under competitive conditions, GITR intrinsically affects GP_33–41_ but not NP_396–404_-specific accumulation, whereas effects of GITR on T cell inhibitory receptor levels are cell-extrinsic, and likely due to increased viral load in the complete GITR-deficient mouse.

### GITR affects the accumulation but not the initial rate of division or the differentiation of LCMV-specific CD4 T cells

**Figure 7:**
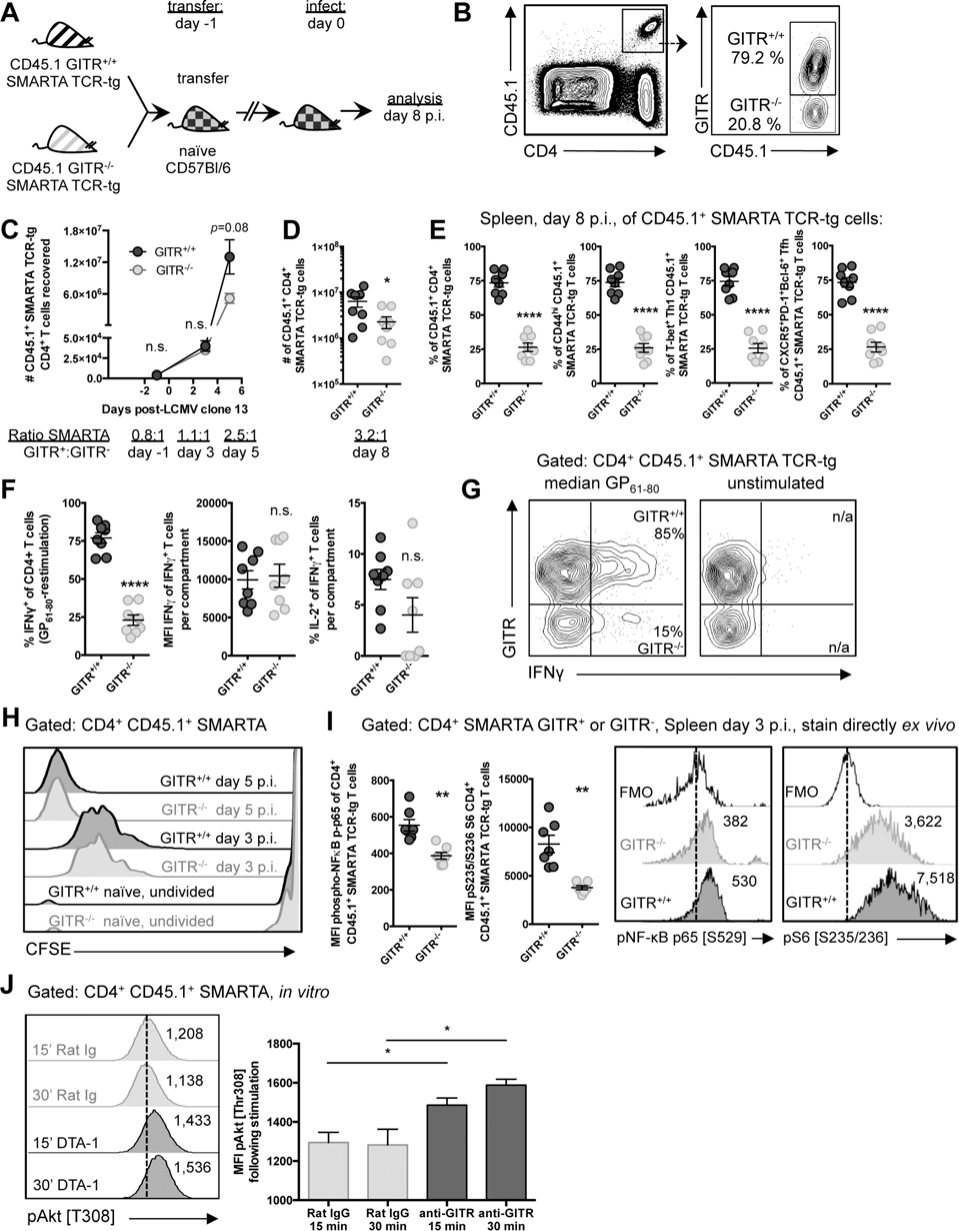
GITR co-stimulation activates classical NF-κB and the Akt-mTORC1 signaling axis to regulate CD4 T cell accumulation post-priming. (A, B) C57BL/6 mice received a 1:1 mixture of GITR^+/+^ and GITR^-/-^ SMARTA, and were infected the following day with LCMV cl 13. At day eight p.i., proportions of GITR^+^ and GITR- cells were evaluated, with gating strategy shown in B. (C) 10^6^ or (D) 10^4^ GITR^+/+^ and GITR^-/-^ CFSE-labeled CD45.1^+^ SMARTA from F2 littermates were co-transferred into naïve CD45.2 C57BL/6 mice one day prior to LCMV cl 13 infection. The total numbers of GITR^+^ and GITR- SMARTA cells in the spleen at different time points following LCMV cl 13 infection are shown. Each symbol in C shows mean ± SEM of at least two to three mice per group, representative of at least two experiments per time point. (E) Proportions of GITR^+^ and GITR- of: total, CD44^hi^, T-bet^+^ Th1, and Tfh SMARTA were evaluated in the spleen at day eight p.i. (F) The proportions of IFNγ^+^ or IFNγ^+^IL-2^+^, and the quantity of IFNγ produced per cell were evaluated in GITR^+^ and GITR^-^ SMARTA CD4 T cells following five hours of GP_61–80_ peptide restimulation, with representative staining shown in G. (H) 10^6^ GITR^+/+^ and GITR^-/-^ CFSE-labeled CD45.1^+^ SMARTA from F2 littermates were co-transferred into naïve CD45.2 C57BL/6 mice. At days three and five p.i., CFSE dilution was evaluated. (I) 10^6^ GITR^+/+^ and GITR^-/-^ CD45.1^+^ SMARTA from F2 littermates were co-transferred into naïve CD45.2 C57BL/6 mice as in A. At day three post-infection, cells were stained directly *ex vivo* for (p) p65 NF-κB pSer529 and (p)S6 ribosomal protein pSer235/236. (J) Activated GITR^+/+^ SMARTA were expanded for two days and then serum starved for 12 hours prior to engaging with 10μg/mL anti-GITR (cone DTA-1) or Rat IgG, followed by evaluation of phosphorylation at Thr308 of Akt. MFI of pThr308 following stimulation is shown, representative of three independent experiments. Numbers adjacent to the representative histograms of I and J are median MFI values. Each symbol in D–G represents an individual mouse, with bars indicating mean ± SEM. Data are pooled from two experiments with a total of eight mice. Data in H show a single representative experiment with two-three mice per group, with at least two independent repeats. Data in I are pooled from two independent experiments with a total of seven mice.

To distinguish between the effects of GITR on CD4 T cell accumulation versus differentiation, we transferred a 1:1 mixture of GITR^+/+^:GITR^-/-^ TCR transgenic SMARTA cells one day prior to LCMV cl 13 infection (Fig. 7A). We used the congenic marker CD45.1 and GITR expression to identify GITR^+/+^ and GITR^-/-^ SMARTA cells within the same mouse (Fig. 7B). Differences in GITR^+/+^ and GITR^-/-^ SMARTA accumulation showed a trend starting at day five p.i., with significant differences by day eight p.i., with a 3.2:1 ratio (Fig. 7, C and D). The ratio of GITR^+/+^:GITR^-/-^ CD44^hi^, Tfh, and Th1 SMARTA cells was uniformly 3:1, indicating that GITR is not skewing the differentiation of particular CD4 effector T cell sub-populations, rather it contributes to the accumulation of all CD4 T cells (Fig. 7E). While there was a lower frequency of GITR- CD4 T cells producing IFNγ after GP_61–80_ peptide restimulation, GITR^+^ and GITR- IFNγ^+^ SMARTA produced a similar amount of IFNγ per cell. There was also a consistent trend toward fewer GITR- IL-2^+^ IFNγ^+^ SMARTA cells relative to the GITR^+^ SMARTA (Fig. 7, F and G).

To determine whether GITR co-stimulation enhanced early division or post-priming accumulation of CD4 T cells, we transferred a mixture of 5×10^5^ each of GITR^+/+^ and GITR^-/-^ CFSE-labeled CD45.1 SMARTA cells into naive C57BL/6 (CD45.2) mice. CFSE dilution at days three and five p.i. was comparable between GITR^-^ SMARTA and GITR^+^ SMARTA (Fig. 7H), suggesting that GITR does not influence initial division, but rather affects post-priming accumulation of CD4 T cells.

### GITR induces NF-κB and mTORC1 activation in LCMV-specific CD4 T cells

The accumulation of T cells during their clonal expansion is well known to require survival signals, such as those mediated by NF-κB, as well as upregulation of protein translation and glycolysis, as mediated by mTOR [37–40]. Therefore, to investigate a possible mechanism underlying the enhanced accumulation of GITR^+^ SMARTA, a 1:1 mixture of GITR^+/+^ and GITR^-/-^ CD45.1 SMARTA were transferred into C57BL/6 (CD45.2) mice and phsopho(p)-p65 NF-κB and (p)S6, a downstream mTOR target were evaluated in SMARTA cells at day three p.i. without further stimulation. Consistent with GITR-dependent NF-κB activation in CD8 T cells [26, 41–43], (p)p65 NF-κB was increased in GITR^+/+^ compared to GITR^-/-^ SMARTA cells at day three p.i. and (p)S6 was also increased, consistent with GITR-dependent mTORC1 activation (Fig. 7I).

mTORC1 can be activated by Akt, a kinase that critically regulates T cell metabolism and expansion [37, 38]. Akt activation has been noted downstream of other TNFRs, such as OX40 [37, 44]. Therefore, we evaluated Akt activation following GITR cross-linking. GITR^+/+^ SMARTA splenocytes were activated *in vitro* with LCMV GP_61–80_ and IL-2 to induce GITR expression, then serum starved for 12 hours, followed by treatment with agonistic anti-GITR (DTA-1) or Rat IgG control. DTA-1 enhanced Akt activation as demonstrated by increased (p)Akt Thr308 at 15 and 30 minutes post-stimulation (Fig. 7J). Taken together, these studies show that GITR intrinsically affects post-priming accumulation but not early division of CD4 T cells with effects on NF-κB and the Akt-mTORC1-S6 signaling axis detected at day three p.i.

## Discussion

The mechanism by which CD4 T cell responses contribute to the control of persistent infection, or how co-stimulation regulates CD4 T cell responses to infection remains incompletely defined [45]. Here we show that GITR directly augments the CD4 Th1 response to LCMV cl 13, which in turn impacts IL-2-dependent CD8 T cell expansion and viral control. The critical role of GITR on the CD4 T cells for control of LCMV cl 13 is demonstrated by the loss of GITR-mediated protection when CD4 T cells are depleted. Signaling downstream of GITR could be detected as early as day three p.i., consistent with an effect of GITR on CD4 T cell accumulation starting around day five and delayed effects on CD8 T cells appearing between days five and eight p.i. These findings are also consistent with strong induction of GITRL by day two p.i. with LCMV cl 13 [46]. These results demonstrate an important role for GITR early in the immune response that impacts post-priming accumulation of CD4 T cells to allow help for CD8 T cells. CD4 T cell-intrinsic GITR also reciprocally affects the Tfh and Tfr responses and, consequently, LCMV-specific IgG production. As Tfh and antibody responses are thought to contribute to the late control of persistent LCMV infection [32], this effect of GITR on Tfh and Tfr likely impacts the late control of the infection rather than the early control, when Tfh and anti-LCMV IgG levels are comparable between GITR^+/+^ and GITR^-/-^ mice.

Previous work demonstrated a CD8 T cell-intrinsic role for GITR on survival of adoptively transferred CD8 T cells during acute respiratory infection of mice with influenza A virus, with no impact on cell division [26]. Here we saw similar effects in the competitive bone marrow chimera experiment (Fig. 6). However, the CD4-depletion studies and the P14 GITR^+/+^ and GITR^-/-^ adoptive transfer studies support a CD8 T cell-extrinsic effect of GITR. LCMV cl 13 infection results in systemic prolonged inflammation, perhaps providing additional signals to compensate for the lack of GITR on CD8 T cells during the early stages of LCMV cl 13 infection. Nonetheless, the effects of GITR on CD8 T cells can be revealed under competitive conditions, but are clearly not sufficient to compensate for the requirement for GITR on the CD4 T cells to allow help for CD8 T cell and antibody responses to chronic LCMV infection.

LCMV-specific CD4 T cells can rescue exhausted CD8 T cell responses to LCMV cl 13, although the precise mechanism of this CD4 T cell helper function remains open to speculation [47]. CD25-deficient CD8 T cells have defective persistence during chronic LCMV infection [30], and IL-2 can be used therapeutically to augment the CD8 T cell response to Armstrong (acute) and cl 13 (chronic) LCMV infection [28, 29]. Here we show that IL-2 blockade in GITR^+/+^ mice resulted in an immune response indistinguishable from that in GITR^-/-^ mice, in which IL-2 blockade had no effect. These findings imply that the effect of GITR on CD4 help for CD8 T cells is IL-2 dependent. Although we have not directly shown that the CD4 T cells are the source of IL-2, taken together with the CD4 depletion studies, the IL-2 blocking data are consistent with a lack of early IL-2^+^ Th1 accumulation as the underlying cause of the defective CD8 T cell accumulation and viral control in GITR^-/-^ mice at day eight p.i.

GITR-deficiency did not result in a change in the proportion or number of Tregs. However, under conditions of competition, there was a two-fold reduction in the number of GITR^-/-^ Tregs, consistent with previous findings that GITR may have a role in peripheral maintenance of Tregs [20]. When CD4 T cells were depleted, the dominant effect was a net increase in viral load, consistent with a loss of T cell help rather than the removal of a regulatory CD4 T cell population in GITR^-/-^ mice. Nonetheless, it is possible that GITR on effector cells could render them resistant to Tregs [20]. Therefore we used DEREG mice to further assess the role of Tregs in our model (S3 Fig.). Diphtheria toxin (DT) was used to deplete Tregs from depletion of regulatory T cells (DEREG) mice, which express the human DT receptor under the *Foxp3* promoter. DT was highly toxic in the LCMV cl 13 model, even in the non-DEREG mice. Viral load in this model was three to four orders of magnitude higher than in non-DT treated GITR^+/+^ mice, making the results difficult to interpret. Moreover, four of 26 LCMV cl 13 infected GITR^+/+^ mice treated with DT died, whereas all GITR^-/-^ mice survived, likely due to reduced levels of inflammatory cytokines. Although the data from these experiments are difficult to interpret given the high level of toxicity, it is clear that Th1 as well as CD8 T cell differences between GITR^+/+^ and GITR^-/-^ mice are largely retained even when >90% of Tregs were depleted. These data argue against a major role for GITR on the CD4 or CD8 effector T cells in preventing the action of Tregs in this model.

Late in LCMV cl 13 infection Tfh accumulate and provide help for antibody responses [32]. GITR^-/-^ mice have impaired Tfh responses with a slight concomitant increase in the Tfr population [35, 36] and a modest decrease in LCMV-specific antibodies. Competitive bone marrow chimeras revealed that GITR also has cell-intrinsic effects on Tfh. GITRL is largely absent on total B cells, whereas a low level of GITRL can be detected on GL-7^+^ Fas^+^ germinal center B cells at days eight and 21 p.i. [46]. It is unlikely that the defect in LCMV-specific IgG production is due to direct effects of GITR on the B cell response, as GITR is largely dispensable for B cell development and activation [48].

Competitive adoptive transfer experiments showed that the absence of GITR on CD4 T cells resulted in a three-fold defect in total LCMV-specific CD4 T cells, Th1, and Tfh sub-populations equivalently, indicating that the Th1 deficit in GITR^-/-^ mice is not due to altered CD4 T cell differentiation. We demonstrated that differences in GITR^+/+^ and GITR^-/-^ CD4 numbers are not due to differences in early CD4 T cell proliferation, but instead due to defects in post-priming accumulation of CD4 T cells. GITR induces classical NF-κB to induce the pro-survival molecule Bcl-x_L_ in CD8 T cells [26]. T cell accumulation also requires upregulation of glycolytic and biosynthetic pathways, such as induced by the Akt-mTORC1 pathway [38]. Here we show that GITR can directly activate Akt and induce phosphorylation of the downstream mTORC1 target S6, as well as activate the classical NF-κB pathway in CD4 T cells as early as day three p.i. Collectively, these signals allow the accumulation of IL-2^+^ Th1 cells, thereby allowing them to help the early CD8 T cell response, as well as to sustain the CD4 response as it progresses towards a Tfh fate.

In sum, we report here a critical CD4 T cell-intrinsic role for GITR in control of chronic LCMV infection. We showed that GITR co-stimulation activates the Akt-mTORC1-S6 signaling axis as well as classical NF-κB signaling to collectively promote CD4 T cell expansion. GITR-deficient mice have defective post-priming accumulation of IL-2^+^ Th1 cells, which precedes an impaired CD8 T cell response and loss of viral control. The effects of GITR on both Th1 and CD8 T cells are IL-2-dependent. We also reveal a novel role for GITR in sustaining the Tfh response and LCMV-specific IgG production. These findings define a critical role for CD4 T cell-intrinsic GITR signaling in early accumulation of CD4 T cells, help for CD8 T cells and viral control.

## Materials and Methods

### Reagents and antibodies

Biotinylated H-2D^b^/GP_33–41_: KAVYNFATM, H-2D^b^/GP_276–286_: SGVENPGGYCL, and H-2D^b^/NP_396–404_: FQPQNGQFI monomers were obtained from the National Institute for Allergy and Infectious Disease tetramer facility (Emory University, Atlanta, GA), and tetramerized with streptavidin APC (Molecular Probes). Anti-CD107a (clone 15A7), anti-CD8 (clone 53–6.7), anti-CXCR5 (clone 2G8), and anti-TNF (clone MP6-XT22) were purchased from BD Biosciences. Anti-phospho-Akt Thr308 (C31E5E) and anti-phospho-S6 ribosomal protein Ser235/236 (clone D57.2.2E) were purchased from Cell Signaling Technologies. Lectin PNA from *Arachis hypogaea* (Cat. #L21409) and viability stain (Cat. #L34955) were purchased from Molecular Probes. Anti-CD4 (clone RM4–5) and CD45.1 (clone A20) were purchased from BioLegend. FITC IgG isotype control, anti-Fas (clone 15A7), anti-CD25 (clone PC61.5), anti-LAP (clone TW7–16B4), anti-PD-1 (clone J43), anti-Tim-3 (clone RMT3–23), anti-ICOS (clone 7E.17G9), anti-CD3 (clone 1245–2C11), anti-CD45.1 (clone A20), anti-CD45.2 (clone 104), anti-CTLA-4 (clone UC10–4B9), anti-B220 (clone RA3–6B2), anti-CD16/32 (clone 93), anti-Foxp3 (clone FJK-16s), anti T-bet (clone eBio4B10), anti-IL-2 (clone JES6–5H4), anti-IFNγ (clone XMG1.2), phospho-NF-κB p65 Ser529 (clone MCFA30), Streptavidin-PE and -APC, and fixable viability dye eFluor 506 (Cat. #65–0866–14) were purchased from eBioscience. Anti-IL-2 (clone S4B6–1) was purchased from Bio X Cell (West Lebanon, NH). Un-nicked Diphtheria Toxin was purchased from List Biological Laboratories, Inc. (Campbell, CA) and was used to deplete Foxp3^+^ Tregs from DEREG mice [49] by administering 1μg i.p. DT per two days from day -2 to day six p.i.

### Mice and LCMV

LCMV Armstrong and cl 13 (obtained from P. Ohashi, University of Toronto and M. Oldstone, Scripps Research institute) were propagated on BHK and L929 cells, respectively (kindly provided by Pamela Ohashi, Princess Margaret Cancer Centre, Toronto, Ontario). GITR^+/+^ and GITR^-/-^ mice were infected intravenously with 2 × 10^6^ ffu of LCMV Armstrong or LCMV clone 13 where indicated. GITR^-/-^ mice were kindly provided by Dr. C. Riccardi (University of Perugia) and Dr. P. Pandolfi (Harvard Medical School). GITR^-/-^ mice were analyzed by SNP analysis across 1500 SNPs differing in the 129 and B6 genome and found to be a minimum of ninety two per cent C57BL/6 (TCAG, University of Toronto). Effects of GITR on CD4 and CD8 T cell effector responses were similar when compared to commercial C57BL/6 mice or when we used F1 or F2 littermate controls. However, the proportion of Tregs in the mice after infection differed in commercial mice versus littermates, therefore all Treg data are reported from experiments with littermate controls. DEREG [49], now available from Jackson Laboratories, P14 [27] and SMARTA [34] mice were obtained from Dr. P. Ohashi (Princess Margaret Hospital, University of Toronto) and additionally crossed to GITR^-/-^ CD45.1 mice and compared with their F2 littermates. All animals were housed under specific pathogen free conditions at the Terrence Donnelly Centre for Cellular and Biomolecular Research (University of Toronto). Where indicated, CD4 T cells were depleted by administering 0.5mg of purified anti-CD4 (clone GK1.5) two days before LCMV cl 13 infection, or IL-2 was blocked by administering 0.5mg anti-IL-2 (clone S4B6–1) given at days four and six p.i. All animal procedures were approved by the animal care committee of the University of Toronto (Protocol approval number: 20009988) in accordance with the Canadian Council on Animal care, a National Regulatory body through which the University of Toronto holds a certificate of good animal care (http://www.ccac.ca/en_/assessment/certification).

### Intracellular cytokine staining

Splenocytes were cultured in complete medium with Brefeldin A and monensin (BD, Cat. #554715) with 1μg/mL D^b^-restricted peptides: NP_396–404_, GP_33–41_, or GP_276–286_ (Anaspec, Fremont, CA) for five hours, or 4μg/mL I-A^b^-restricted GP_61–80_ with 20U/mL rmIL-2 (eBioscience) for five hours. Cells were then surface stained, fixed and permeabilized (BD), and stained for intracellular cytokine production.

### LCMV focus forming assay

Organs were harvested and immediately placed on dry ice. Organs were later thawed, homogenized, and supernatant dilutions (range: 10^0–^10^5^) were used to infect an MC57 cell monolayer under a 2% methylcellulose-MEM overlay. 48 hours later, monolayers were fixed with 4% PFA, permeabilized with 1% Triton X-100, and stained with Rat anti-LCMV mAb (clone VL-4). Following secondary Goat anti-Rat-HRP, a colorimetric reaction with *o*-Phenylenediamine (Sigma-Aldrich) was used to quantify LCMV-infected foci.

### LCMV-specific IgG quantification

LCMV-specific IgG ELISAs were performed as previously described [50], with modification to the purification of LCMV. LCMV was purified on a CsCl gradient of 1.45g/L and 1.2g/L in 10mM Tris (pH 8.1). Plates were coated with 10ng of inactivated purified LCMV cl 13 and blocked with Superblock in TBST (Thermo Fisher Scientific), followed by one-hour incubation with dilutions of plasma from infected animals. 1:2,000 HRP-tagged anti-mouse secondary antibody was then added and incubated for one hour. 3,3’,5, 5’-tetramethylbenzidine (Sigma Aldrich) was used for colorimetric reaction and absorbance was measured at 456nm.

### Adoptive transfer-based assays and bone marrow chimeras

Purification of CD8 T cells for P14 adoptive transfers or CD4 T cells for SMARTA adoptive transfers were achieved with negative selection kits according to manufacturer’s protocols (StemCell Technologies, Vancouver, BC, Cat. 19753A and 19752, respectively). For P14 and SMARTA transfers, cells were transferred intraperitoneally one day prior to LCMV infection, and where indicated, were labeled with 10μM Carboxyfluorescein Diacetate Succinimidyl Ester (CFSE). GITR^+/+^: GITR^-/-^ mixed bone marrow chimeras were generated by intravenously reconstituting lethally irradiated C57BL/6 mice with a 1:1 mixture of GITR^-/-^ CD45.1: GITR^+/+^ CD45.2 bone marrow cells for a total of 5 × 10^6^ cells. Following irradiation and reconstitution, mice were given 2mg/mL neomycin sulfate (Bio-Shop, Burlington, ON) for two consecutive weeks, after which mice were rested for an additional 90 days before use.

### Phosphoflow assays

For *in vitro* studies, SMARTA splenocytes were stimulated with 4μg/mL I-A^b^-restricted GP_61–80_ with 40U/mL recombinant murine IL-2 in complete RPMI containing 10% FCS for two days. Cells were rested overnight in serum-free Aim V medium with 1X β-Mercaptoethanol (Invitrogen). Live SMARTA cells were stimulated with 10μg/mL anti-GITR (DTA-1, hybridoma provided by S. Sakaguchi, Osaka University) or Rat IgG isotype control (Jackson) each followed by 5μg/mL Goat anti-Rat (Jackson). Samples were prepared for flow cytometry using BD Phosflow Buffer I (BD, Cat. #557870). Similar protocols were used for direct *ex vivo* staining of freshly isolated splenocytes at day three p.i.

### Data analysis and statistics

Samples were acquired with FACS Canto, LSR II, or LSR Fortessa (BD Biosciences) with FACSDiva software. Flow cytometry data were analyzed with FlowJo v9 (Tree Star, Inc., Ashland, OR). All statistical analyses were performed using GraphPad Prism v6 (La Jolla, CA). Unpaired Student’s *t*-tests were used to compare two groups, with *P* values indicated on figures: * *P* < 0.05, ** *P* < 0.01, *** *P* < 0.001, **** *P* < 0.0001.

## Supporting Information

S1 Fig CD8 T cell surface and intracellular cytokine stain gating strategy. Freshly isolated mouse peripheral blood mononuclear cells or splenocytes were used for flow cytometry. Numbers in bold indicate hierarchical populations. Gating was first based on physical properties: (1) lymphocytes; (2) singlets; followed by gating on (3) live cells; (4) CD3^+^CD8^+^ T cells. From the CD3^+^CD8^+^ gate, strategies differ based on “surface” or “intracellular stain.” Surface: tetramers were gated on from 4 (based on naïve controls) and from the (5a, b) tetramer^+^ gate, (6a) levels of PD-1, Tim-3, and others were determined. Intracellular: IFNγ^+^ cells were gated on from 4 (see unstimulated control). From the (5c) IFNγ^+^ gate, (6b) polyfunctional TNF^+^ CD107a^+^ were identified using FMOs. Representative data are from day eight p.i. spleens.
(TIF)

S2 Fig CD4 T cell surface and intracellular stain gating strategy. Freshly isolated mouse peripheral blood mononuclear cells or splenocytes were used for flow cytometry. Bolded numbers indicate hierarchical populations. Gating was first based on physical properties: (1) lymphocytes; (2) singlets; followed by (3) live cells and (4) CD3^+^CD4^+^ T cells. From the CD3^+^CD4^+^ gate, strategies differ based on surface or intracellular stain. Surface: from 4, cells were identified as 5a Tfh (CXCR5^+^PD-1^+^, then sub-gated to see that these cells were ICOS^hi^ Bcl-6^hi^ Foxp3^-^; Foxp3^+^ Tfh were defined as Tfr (6a). Note: Bcl-6 and ICOS were not included in every experiment, but have been used in at least two experiments to ensure that the PD-1^+^CXCR5^+^ cells are Tfh. From 4, cells were also stained for CD44 and T-bet (5b, Th1) and Foxp3 and CD25 (5c, Treg). Intracellular cytokine staining: IFNγ^+^ cells (5d) were gated on from 4. From the 5d IFNγ^+^ gate, (6b) co-production of IL-2 was determined based on FMO controls. Data shown are from spleens at various time points.
(TIF)

S3 Fig GITR-deficient Tregs do not play a critical role in the impaired immunity of GITR^-/-^ mice to LCMV cl 13. (A) Experimental design for the depletion of Tregs: GITR^-/-^ DEREG or non-DEREG and GITR^+/+^ DEREG or non-DEREG F2 littermates were treated with 1μg diphtheria toxin i.p. at days -2, 0, 2, 4 and 6 p.i. to deplete Tregs for the first seven days of LCMV cl 13 infection. (B) Raw and summary data showing efficiency of Treg depletion from spleen at day seven p.i. (C, D) The absolute numbers of CD4^+^ T-bet^+^ Th1 and D^b^/NP_396–404-_ and D^b^/GP_33–41_-specific CD8^+^ T cells are shown in the spleen from day seven p.i. (E) Viral load in the spleen and kidney at day seven p.i. Data are pooled from two experiments with a total of at least five mice per group. Note: DT is toxic in the LCMV cl 13 model, even in the non-DEREG mice, resulting in a viral load that is three to four orders of magnitude higher than non-DT-treated GITR^+/+^ mice (Fig. 1A) making viral load difficult to interpret in this experiment. Four of 26 GITR^+/+^ and 0 of 11 GITR^-/-^ LCMV cl 13 infected mice died from simultaneous DT treatment.
(TIF)
