# Supplementary figures and images for: GITR Intrinsically Sustains Early Type 1 and Late Follicular Helper CD4 T Cell Accumulation to Control a Chronic Viral Infection
Source: PLoS Pathog. 2015 Jan 15;11(1):e1004517. doi: 10.1371/journal.ppat.1004517 (PMC4295864; doi:10.1371/journal.ppat.1004517)

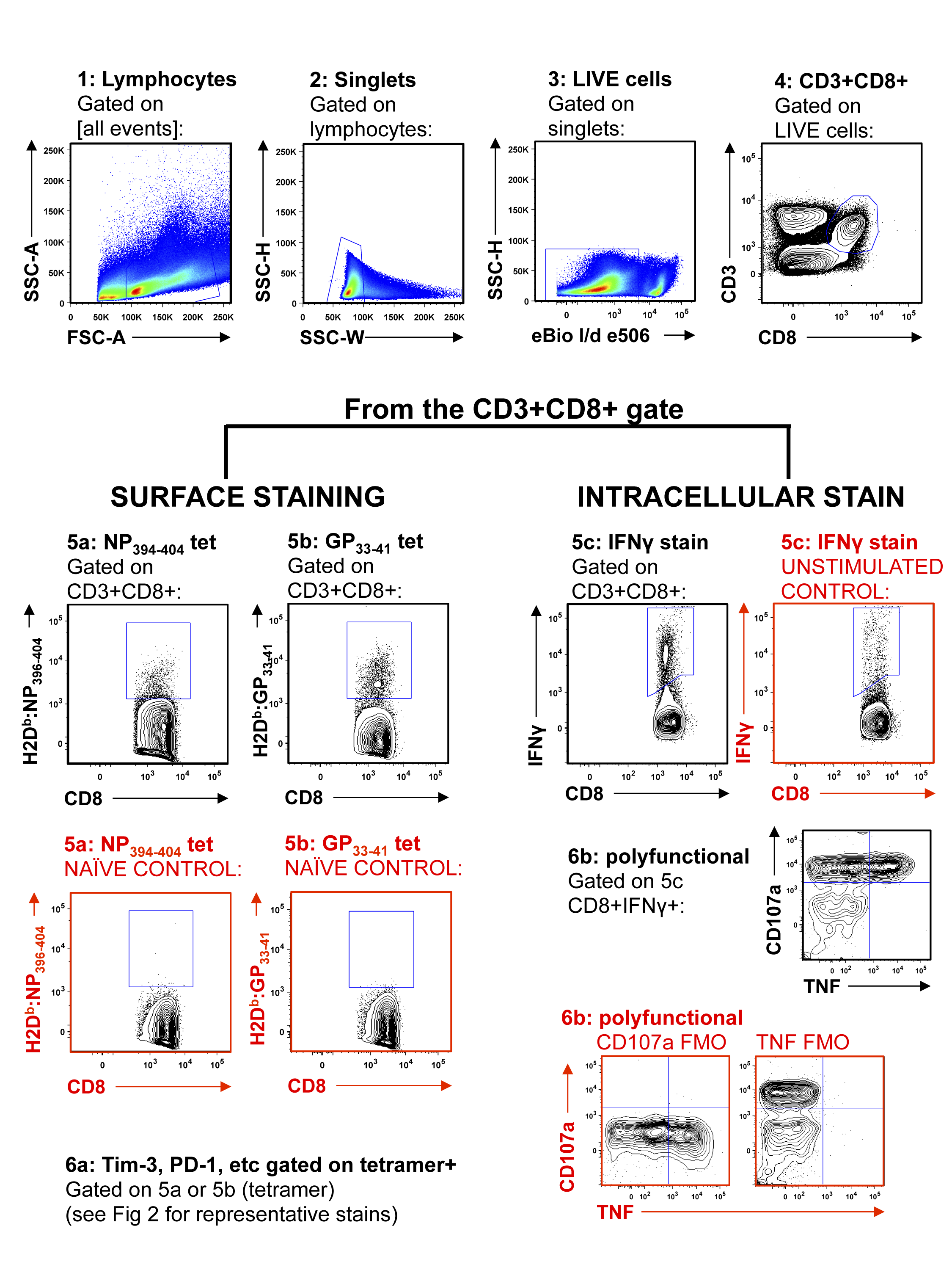

Supplement: S1 Fig — Freshly isolated mouse peripheral blood mononuclear cells or splenocytes were used for flow cytometry. Numbers in bold indicate hierarchical populations. Gating was first based on physical properties: (1) lymphocytes; (2) singlets; followed by gating on (3) live cells; (4) CD3+CD8+ T cells. From the CD3+CD8+ gate, strategies differ based on “surface” or “intracellular stain.” Surface: tetramers were gated on from 4 (based on naïve controls) and from the (5a, b) tetramer+ gate, (6a) levels of PD-1, Tim-3, and others were determined. Intracellular: IFNγ+ cells were gated on from 4 (see unstimulated control). From the (5c) IFNγ+ gate, (6b) polyfunctional TNF+ CD107a+ were identified using FMOs. Representative data are from day eight p.i. spleens. (TIF) [file ppat.1004517.s001.tif]

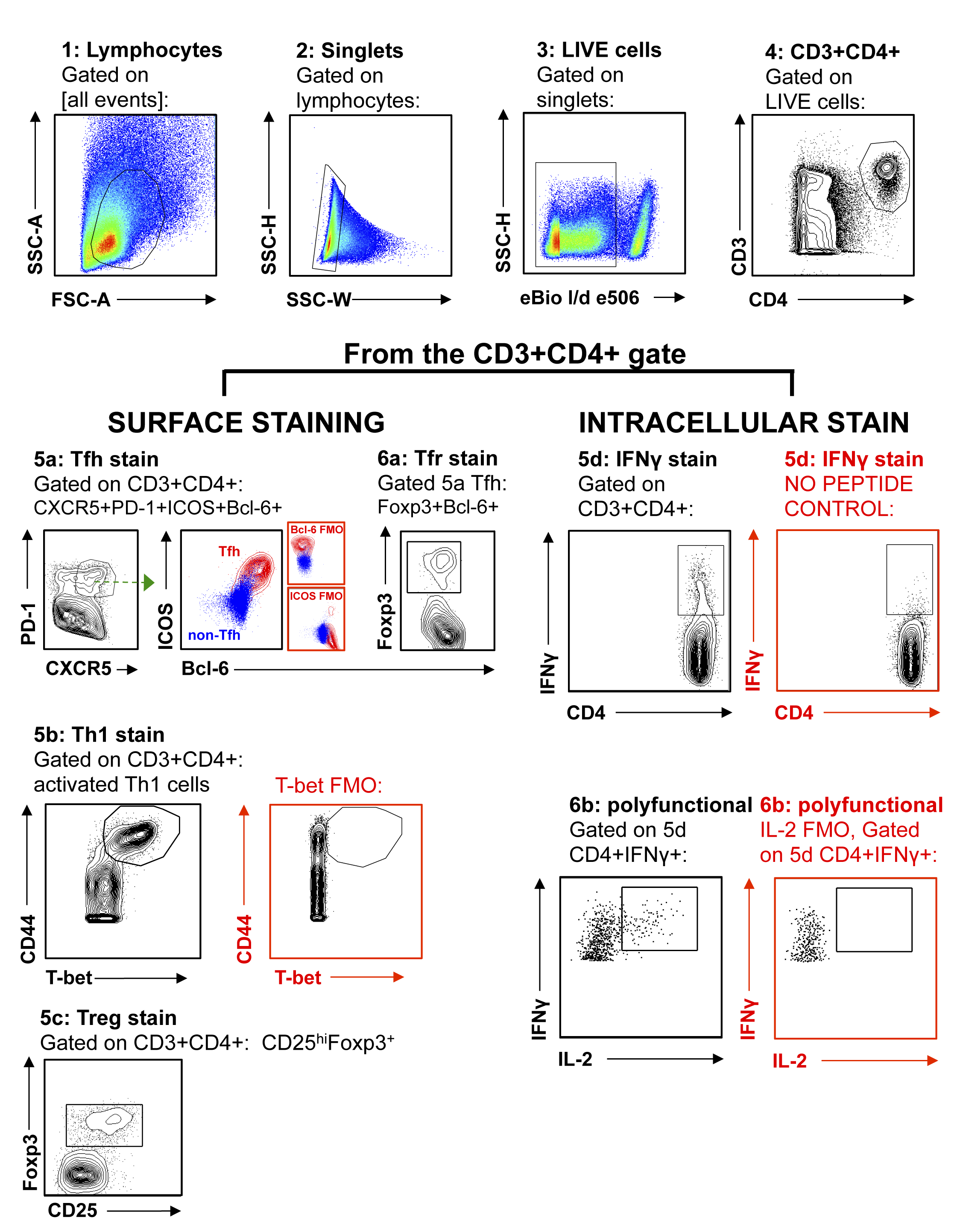

Supplement: S2 Fig — Freshly isolated mouse peripheral blood mononuclear cells or splenocytes were used for flow cytometry. Bolded numbers indicate hierarchical populations. Gating was first based on physical properties: (1) lymphocytes; (2) singlets; followed by (3) live cells and (4) CD3+CD4+ T cells. From the CD3+CD4+ gate, strategies differ based on surface or intracellular stain. Surface: from 4, cells were identified as 5a Tfh (CXCR5+PD-1+, then sub-gated to see that these cells were ICOShi Bcl-6hi Foxp3-; Foxp3+ Tfh were defined as Tfr (6a). Note: Bcl-6 and ICOS were not included in every experiment, but have been used in at least two experiments to ensure that the PD-1+CXCR5+ cells are Tfh. From 4, cells were also stained for CD44 and T-bet (5b, Th1) and Foxp3 and CD25 (5c, Treg). Intracellular cytokine staining: IFNγ+ cells (5d) were gated on from 4. From the 5d IFNγ+ gate, (6b) co-production of IL-2 was determined based on FMO controls. Data shown are from spleens at various time points. (TIF) [file ppat.1004517.s002.tif]

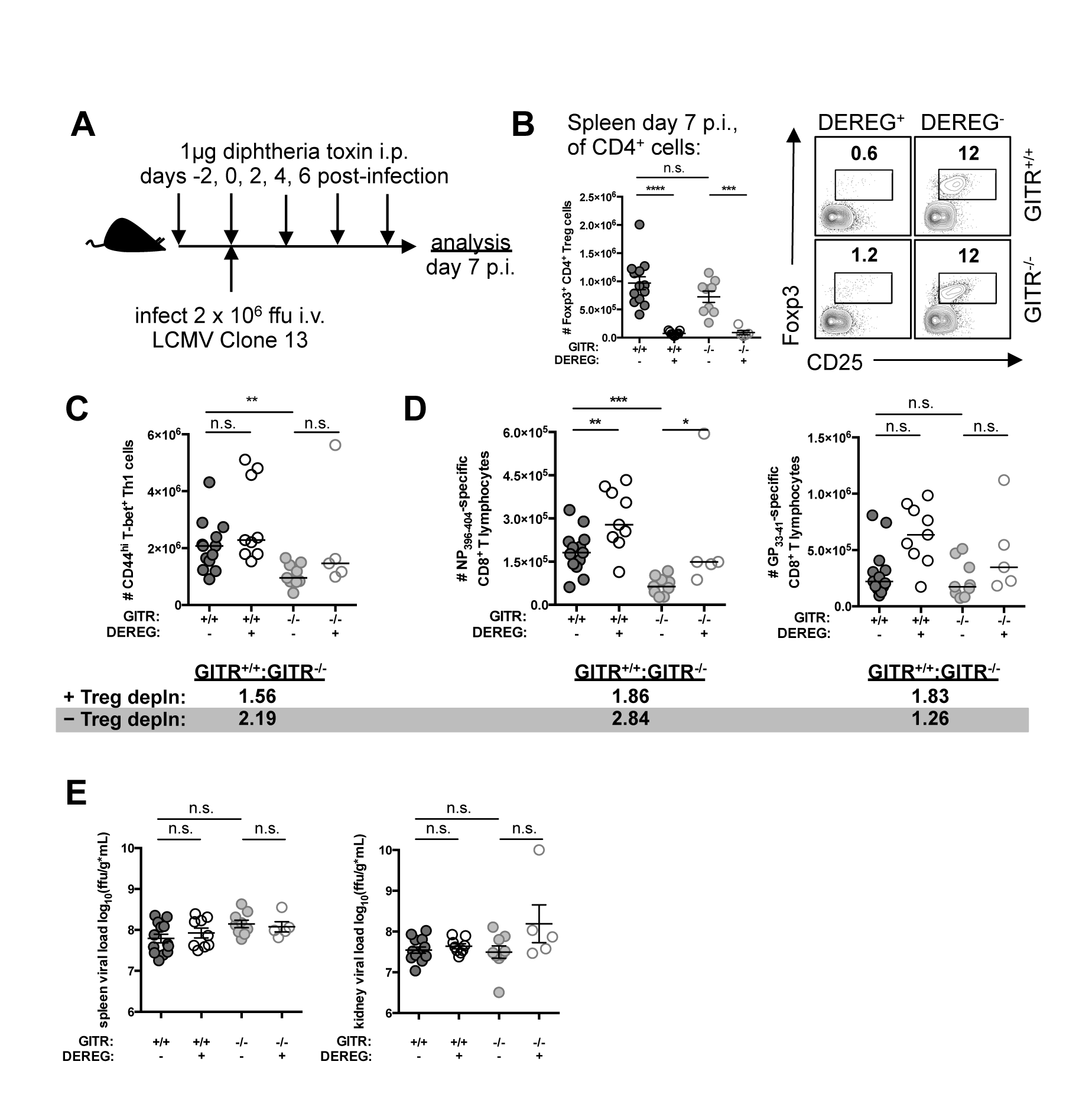

Supplement: S3 Fig — (A) Experimental design for the depletion of Tregs: GITR-/- DEREG or non-DEREG and GITR+/+ DEREG or non-DEREG F2 littermates were treated with 1μg diphtheria toxin i.p. at days -2, 0, 2, 4 and 6 p.i. to deplete Tregs for the first seven days of LCMV cl 13 infection. (B) Raw and summary data showing efficiency of Treg depletion from spleen at day seven p.i. (C, D) The absolute numbers of CD4+ T-bet+ Th1 and Db/NP396–404- and Db/GP33–41-specific CD8+ T cells are shown in the spleen from day seven p.i. (E) Viral load in the spleen and kidney at day seven p.i. Data are pooled from two experiments with a total of at least five mice per group. Note: DT is toxic in the LCMV cl 13 model, even in the non-DEREG mice, resulting in a viral load that is three to four orders of magnitude higher than non-DT-treated GITR+/+ mice (Fig. 1A) making viral load difficult to interpret in this experiment. Four of 26 GITR+/+ and 0 of 11 GITR-/- LCMV cl 13 infected mice died from simultaneous DT treatment. (TIF) [file ppat.1004517.s003.tif]
